# Optical levitation of Janus particles within focused cylindrical vector beams

**DOI:** 10.1515/nanoph-2024-0774

**Published:** 2025-04-28

**Authors:** Alessandro Magazzù, Iryna Kasianiuk, Denis Kasyanyuk, Agnese Callegari, Giovanni Volpe, Onofrio M. Maragò, Luca Biancofiore

**Affiliations:** CNR-IPCF, Istituto per i Processi Chimico-Fisici, I-98158, Messina, Italy; Department of Mechanical Engineering, Bilkent University, TR-06800, Ankara, Türkiye; UNAM – National Nanotechnology Research Center and Institute of Materials Science & Nanotechnology, Bilkent University, 06800, Ankara, Türkiye; Department of Physics, University of Gothenburg, SE-41296, Gothenburg, Sweden; Department of Industrial and Information Engineering and Economics, University of L’Aquila, L’Aquila, Italy

**Keywords:** Janus particle, optical levitation, cylindrical vector beams

## Abstract

The confinement and manipulation of Janus particles have recently garnered significant interest due to their potential applications in fields such as nanotechnology and biophysics, where, under specific circumstances, they can act as microengines and drug carriers. However, the dynamics of Janus particles mostly rely on chemical reactions or thermal gradients, limiting their precision application. To tackle these limitations, we propose the 3D manipulation of Janus particles using focused cylindrical vector beams with a doughnut shaped intensity profile above the focal spot. In particular, we study the behaviour, orientation and manipulation of different highly reflective Janus particles composed of silica or polystyrene with a gold cap in the presence of optical potentials generated by focused cylindrical vector beams. Where the radiation pressure predominantly affects the gold cap rather than the bare particle body of the particle. We demonstrated the potential of the proposed levitation technique for controlling a wide range of Janus particles and real-life complex objects with high reflectivity.

## 1 Introduction

Federico Capasso’s pioneering research in optical physics, particularly in the fields of photonics and optical forces [1], has contributed to advancements in optical manipulation techniques [2]. His work on structured light, including the development of metasurfaces and vector beams, has opened new avenues for precise control over light–matter interactions at the microscale [3], [4], [5]. Capasso’s research on tailored optical fields has contributed to innovations in optical trapping, enabling novel approaches to manipulate particles with high spatial and force resolution [2].

The concept of light exerting a force on matter traces back to the 17th century when Johannes Kepler proposed that the dust tail of a comet is a result of the radiation pressure exerted by the Sun’s rays on sublimated components of the comet. Radiation pressure refers to the mechanical pressure exerted upon any surface due to the exchange of momentum between photons and matter. Despite the momentum carried by photons being extremely small, interest in radiation pressure and its applications surged following the invention of the laser. Building on the pioneering experiments by Arthur Ashkin and coworkers on optical forces and optical trapping [6], [7], [8], [9], optical tweezers (OT) have become a powerful tool for the contactless manipulation of micro and nano objects by a highly focused laser beam via a high numerical aperture (NA) objective [10], [11], [12], [13].

In this study, we utilise optical forces generated by structured light fields, as explored in Capasso’s work and others [3], [4], [5], [14], [15], [16], providing stable confinement and control over reflective Janus particles. These particles, named after the Roman god Janus who is depicted with two faces looking in opposite directions, refer to asymmetric colloidal particles with two distinct physical or chemical properties on their opposing hemispheres. These unique structures have garnered significant attention in various scientific disciplines due to their remarkable properties and versatile applications. Originally proposed by Pieranski [17] in 1980 the importance of Janus particles was first addressed by the Nobel Laureate P. G. de Gennes in his Nobel lecture [18] entitled “Soft Matter” in 1991. Indeed, Janus particles have since become a focal point of research in fields such as materials science, nanotechnology, colloid chemistry, and biophysics [19], [20].

The asymmetric nature of Janus particles enables them to exhibit intriguing behaviours and functionalities that are not achievable with homogeneous particles. Their asymmetry can arise from differences in surface chemistry, composition, morphology, or physical properties such as polarity, wettability, or charge distribution. As a result, Janus particles possess distinctive properties, including directional self-propulsion, selective adsorption, controlled assembly, and responsive behaviour to external stimuli [19], [21], [22], [23], [24]. The broken symmetry of Janus particles give them the ability to undergo self-propelled motion in response to external stimuli, such as chemical gradients, light, temperature gradients, or electric fields. This unique capability has led to their exploration as artificial microswimmers for applications in drug delivery, environmental remediation, and microengines [19], [23], [25], [26].

Traditional methods for manipulating Janus particles, such as those relying on chemical or thermal gradients, face several critical limitations. These approaches often lack the fine spatial and directional control required for precise manipulation, making them unsuitable for tasks demanding high accuracy. This limitation is particularly evident in techniques based on chemical or thermal gradients, which are inherently imprecise and constrained in their applicability to complex systems. Furthermore, Janus particles with highly reflective metal caps, such as gold, pose significant challenges for optical trapping and manipulation due to the scattering forces they generate. These challenges can be overcome by harnessing focused vector beams, which enable the application of precise optical forces and torques on Janus particles [27]. This provides a unique opportunity to engineer complex assemblies and structures, thereby expanding the design possibilities for the application of Janus particles in advanced devices [24]. In this study, we investigate the behaviour, orientation, and manipulation of Janus particles composed of silica (SiO_2_) or polystyrene (PS) with gold caps, in the presence of optical potentials generated by focused cylindrical vector beams (CVBs). Our approach achieves three-dimensional optical levitation and control of Janus particles, effectively addressing the current limitations in their 3D manipulation.

## 2 Theoretical background

A Janus particle immersed in an optical field generated by a focused laser beam is primarily subjected to optical forces and thermal effects, resulting from the scattering and absorption of incident photons, predominantly at its cap rather than the bare SiO_2_ particle body.

### 2.1 Optical forces

A full understanding of optical forces requires the full electromagnetic theory describing the light–matter interaction based on the Maxwell’s equations [10], [28]. However, some simplifications and approximations, depending on the particle size can be made to provide an easier understanding and physical insight of optical forces [10], [29], [30], [31]. When the particle size is much smaller than the wavelength *λ* of the laser beam, the size parameter *x*_*p*_ = *πdn*_*m*_/*λ* ≪ 1, it is possible to use the dipole approximation [10], [29], where *d* is the particle diameter, and *n*_*m*_ is the refractive index of the medium surrounding the particle. In this approximation the particle is considered an induced dipole immerse in the electric field generated by the focused laser beam. When the particle size increases and becomes comparable to *λ*, *x*_*p*_ ∼ 1, it is possible to calculate the optical forces using Mie theory or T-matrix methods [29], [32], [33], [34], this is particularly relevant for 0.1 < *x*_*p*_ < 10 [33]. When *x*_*p*_ ≫ 1, as in our experiments (*x*_*p*_ ≈ 26), the applicability of the Mie theory or T-matrix methods can become computationally intensive, and the use of geometrical optics (GO) approximation is more efficient [35], [36]. In this case, the incoming optical field, generated by the focused laser beam, can be considered as a collection of light rays carrying a portion of the total optical power and linear momentum.

When a ray impinges on a particle, it will be partly transmitted and partly reflected, according to the Snell’s law. During these events, a certain amount of momentum $\Delta \vec{P}={\vec{P}}_{\text{inc}}-{\vec{P}}_{\text{ref}}$ is exchanged between the ray and the particle, where *P*_inc_ and *P*_ref_ are the momenta associated with the incident and outgoing rays. The exchanged momentum $\Delta \vec{P}$ in the time interval Δ*t* generates a total optical force $\vec{F}=\frac{\Delta \vec{P}}{\Delta t}$ . This optical force, depending on experimental conditions (e.g., refractive index of the medium and particle, NA objective) can attract the particle toward the focus spot or pushing it away from it [29], [37]. The optical force can be calculated from the scattering, summing the contribution of the force due to the single rays [38], [39]:

(1) $$F_{\text{opt}}=\underset{m}{\sum }F_{\text{ray}}^{\left(m\right)}$$

with

(2) $$F_{\text{ray }}=\frac{n_{m}P_{i}}{c}\hat{i}-\frac{n_{m}P_{r}^{\left(1\right)}}{c}{\hat{r}}_{1}-\overset{\infty }{\underset{j=2}{\sum }}\frac{n_{m}P_{t}^{\left(j\right)}}{c}{\hat{t}}_{j},$$

where *n*_*m*_ is the refractive index of the medium, *c* is the speed of light, $\hat{i}$ , $\hat{r}$ and $\hat{t}$ are unit vectors representing the direction of the incident, reflected and transmitted rays, respectively and *P*_*i*_, *P*_*r*_ and *P*_*t*_ are the incident, reflected and transmitted power respectively.

In the case of a laser beam focused by a low NA objective on a highly reflective Janus particle, the scattering forces will be stronger than the gradient forces, pushing the particle toward the laser beam propagation direction. The scattering forces due to radiation pressure depend primarily on the thickness of the gold cap and under normal incidence can be expressed as:

(3) $$F_{rp}\left(h\right)=\frac{P_{i}}{c}\left(2R\left(h\right)+A\left(h\right)\right),$$

where *R*(*h*) and *A*(*h*) are the reflectance and the absorbance respectively, which depends on the gold cap thickness *h*, see Supplementary Information for more details. [40]. The direction of radiation pressure repulsion is strongly dependent on where the light impinges on the Janus particle which is also influenced by the orientation of its metallic cap typically pointing downwards due to gravity. In the case when the cap points downwards, for normal incident of the beam, the optical force pushes the Janus particle upwards, whereas for different incident angles, the cap causes the optical force direction to shift radially outward from the beam centre [24].

### 2.2 Cylindrical vector beams

Enhancement and customization of optical forces can be achieved through careful selection of the trapping and levitation beam properties, such as intensity profile and polarisation state. Specifically, shaped or structured light beams, like cylindrical vector beams (CVBs), can provide unique optical trapping and levitation effects [41], [42]. CVBs are general vectorial solutions to the Helmholtz equation for beams having both the electric and magnetic fields with cylindrical symmetry in the transverse plane and complex polarisation distributions.

**Figure 1:**
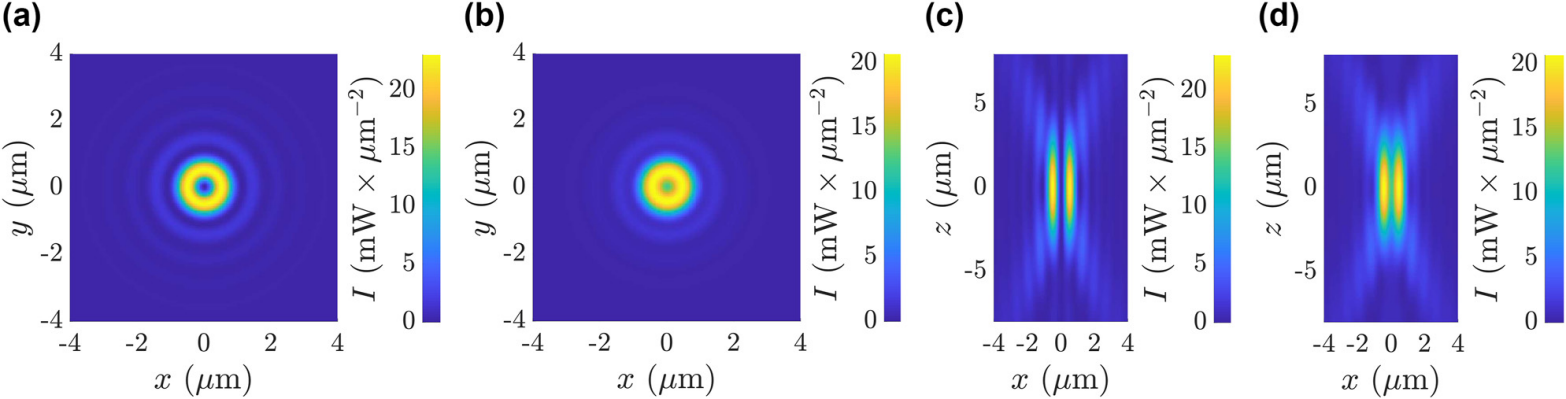
Intensity profiles of the focused CVB around the focus in the case of: (a) azimuthal polarisation and (b) radial polarisation with the sections of planes perpendicular to the propagation axis (*z*-axis). (c) Azimuthal polarisation and (d) radial polarisation with the sections of planes passing through the propagation axis. Here the colour bar for the intensity is represented separately for each figure and the intensity profiles are calculated for NA = 0.7, *λ* = 976 nm and *n*_*m*_ = 1.33.

A special subset of CVBs are the ones obtained as superposition of Laguerre–Gaussian (LG) beams, where the polarisation is simpler, such as azimuthal or radial polarisation, and when considering scalar (rather than vectorial) field descriptions [43], [44] [45], [46]. In the azimuthal polarisation case, the electric field vectors are oriented tangentially around the beam axis, forming concentric circles in the transverse plane as shown in Figure 1(a). This configuration leads to a zero longitudinal field component and a stronger transverse field component around the focus, resulting in a doughnut-shaped intensity profile with high contrast, as shown in Figure 1(a) and (c) [41], [47]. In contrast, for radial polarisation the electric field is directed radially outward from the beam axis. The polarisation vector at each point on the transverse plane points away from the centre, forming, also in this case, concentric rings of electric field vectors. When tightly focused, radially polarised beams tend to produce a stronger longitudinal field component along the optical axis, with a comparatively weaker transverse field component, generating a high-intensity spot at the focus, as shown in Figure 1(b) and (d).

### 2.3 Gravity

Whenever a Janus particle is immersed in a liquid, we also have to consider buoyancy, a force pointing upward contrasting the gravity force *F*_grav_, respectively [39]:

(4) $$F_{grav}=\frac{\pi }{6}d_{JP}^{3}g\left(\frac{\rho_{cap}V_{cap}+\rho_{bare}V_{bare}}{V_{total}}\right)$$

and

(5) $$F_{buoy}=\frac{\pi }{6}\rho_{W}d^{3}g,$$

where *ρ*_cap_, *ρ*_bare_ and *ρ*_W_ are the densities of the cap, bare particle, and water, respectively; *V*_cap_ and *V*_bare_ are the volumes of the cap and the bare particle, respectively; *d* is the particle diameter and *g* is the gravitational acceleration. When Janus particles are illuminated by light, heat is generated due to the absorption of light by the metallic part of the particle and the surrounding medium.

### 2.4 Thermal forces

Due to the extremely high thermal conductivity of gold, heat distributes almost instantaneously across the cap, minimizing internal temperature gradients. As a result, the gold cap can be considered isothermal, and the temperature increase can be computed as [26], [48], [49]

(6) $$\Delta T_{cap}=\frac{P_{\text{abs}}}{\left(\pi +2\right)\kappa_{m}d},$$

where *κ*_*m*_ is the thermal conductivity of the medium and *P*_abs_ is the absorbed power and the term (*π* + 2) is a geometrical factor arising from the heat diffusion model around a hemisphere (the gold cap) embedded in a spherical geometry. The temperature increase Δ*T*_cap_ generates a temperature gradient in the fluid around the particle

(7) $$\nabla T=\frac{2\Delta T_{cap}}{\pi d}.$$

This gradient creates an imbalance in molecular interactions at the particle surface, leading to localized fluid flow along the particle’s surface. This surface flow corresponds to the slip velocity *v*_slip_ = −*D*_*T*_ ∇*T*, where *D*_*T*_ is the thermophoretic mobility, which quantifies how particles respond to a temperature gradient [48], [49]. This flow is driven by the differential temperature across the particle’s surface inducing a thermophoretic force on the Janus particle

(8) $$F_{\text{th}}=\frac{k_{B}T}{D}v_{\text{slip}},$$

where *D* is the diffusion constant. This force is assumed to push the particle in the direction from its coated cap to its uncoated end, i.e., from the hot to the cold region [26].

Due to gravity, the gold cap of the Janus particles always points downward. When illuminated by light, it heats up, generating thermal forces that push the particle upward. These thermal forces, together with radiation pressure, contribute to the levitation of the particle, with the equilibrium position determined by the balance among gravity, radiation pressure, and thermal forces. In our experiments, 6 μm SiO_2_ JPs with a 50 nm thick gold cap were levitated by a laser beam with a power of 50 mW. Under these conditions, the particles reached an equilibrium position approximately 50 µm above the focal spot. At this height, each Janus particle absorbed approximately 6.4 μW resulting in a temperature increase of the gold cap by Δ*T*_cap_ = 0.35 K, see Supplementary Information for more details. It is important to note that, at this elevation, the heating is relatively modest and does not significantly affect the viscosity of the surrounding liquid. In contrast, when the same laser power (50 mW) is applied at a distance of *z* = 1 μm from the focus, the temperature increase is substantially higher, reaching Δ*T*_cap_ = 54.02 K. This significant heating alters the properties of the surrounding medium and enhances the thermal forces that push the particle upward. As the distance from the focal point increases toward the equilibrium position, the beam intensity and thus the absorbed power, temperature increase, and thermal forces decrease. For instance, at *z* = 10 μm, the temperature rise is Δ*T*_cap_ = 8.72 K while *z* = 15 μm, it is reduced to Δ*T*_cap_ = 2.24 K.

It is noteworthy that thermophoresis is a complex phenomenon with many possible theoretical explanations. Here, we report the model we used, which is not the only possible one [50], [51].

## 3 Materials and methods

### 3.1 Manufacturing of Janus particles

**Figure 2:**
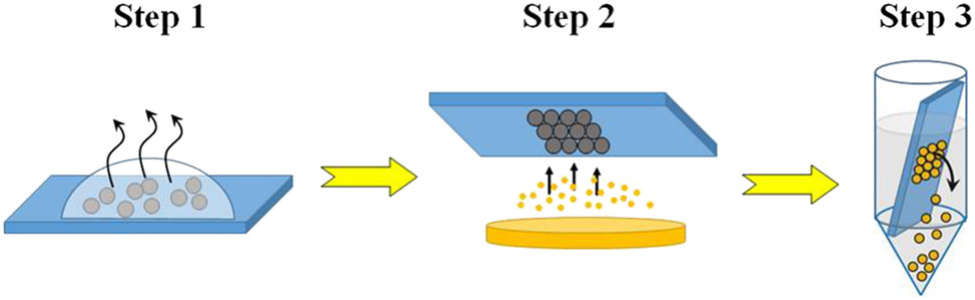
Pictorial representation of basic steps required for fabrication of spherical half-coated Janus particles with metallic cap.

In our work, we used two types of Janus particles based on silica (SiO_2_) or polystyrene (PS) spheres, each with diameters of 6 μm, featuring a gold-coated hemisphere. The fabrication of these particles involves three main steps: preparing a close-packed monolayer of microparticles on a substrate; half-coating the spheres with a metallic layer using a thermal evaporator; and finally, detaching and dispersing the half-coated spheres into an aqueous medium, as illustrated in Figure 2. This process results in a suspension of high-quality dielectric spheres with a reflective metallic cap covering half of them, while the opposite side remains pristine, forming the characteristic Janus faces.

The fabrication of half-coated Janus spheres begins by forming a highly ordered monolayer of silica or polystyrene spheres on a glass slide. To prevent microparticles from sticking, hydrophilic glass slides are used, which are pre-treated with a 0.25 M NaOH solution for 5 min. Droplets of particle suspension are then gently applied to the glass substrate. Simple calculations are conducted beforehand to determine the required particle concentration based on droplet size and particle diameter to create a uniform colloidal monolayer over a defined area. After depositing the droplets, solvent evaporation occurs, allowing the microparticles to self-organize into a periodic lattice. To improve the quality of the final crystalline structure, the evaporation rate is reduced by covering the substrate with an upside-down Petri dish and maintaining a stable temperature of 19 °C.

After the substrates dries completely, thermal evaporation is used to coat them with a metallic layer, as this technique allows fine control over metal layer thickness and produces a high-quality, smooth deposition. This approach involves condensing evaporated metal atoms onto the particle surfaces under high vacuum conditions with a thickness variability of ±10 %. Gold is selected as the primary coating material due to its low reactivity and high stability, while a thin titanium layer was applied first to improve the adhesion of gold to the particle surfaces. Janus particles with a 2 nm thick titanium cap and a 50–140 nm of gold cap were produced. This range of cap thicknesses was chosen to achieve high reflectivity (>98 %), thereby excluding the levitation of microparticles through light refraction (as in the classical optical tweezers technique). To detach the half-coated silica or polystyrene particles from the glass surface, the substrates is placed in Falcon tubes and sonicated for 4–5 s. The tubes is then centrifuged at 3,000 rpm for 5 min, allowing the Janus particles to settle at the bottom. Finally, the glass slide is removed from the solution, leaving a suspension of Janus particles. The concentration of the final solution is controlled by estimating the ratio between the number of particles in the produced monolayers and the volume of distilled water added to the Falcon tube.

### 3.2 Samples preparation

The Janus particles dispersed in distilled water at a concentration of few particles/μl are confined in sandwich-type cells composed of two microscope glass slides separated by spacer strips. The glass slides are decontaminated sequentially with ethanol, acetone, and isopropanol, followed by a 6-min treatment in a 0.25 M NaOH solution to enhance substrate hydrophilicity and prevent particle adhesion. The internal cell thickness is maintained uniformly at 130 μm using a Mylar spacer film. The filled cells are sealed around the edges with fast-setting epoxy glue to prevent external influences and eliminate flux caused by water evaporation.

### 3.3 Experimental setup

**Figure 3:**
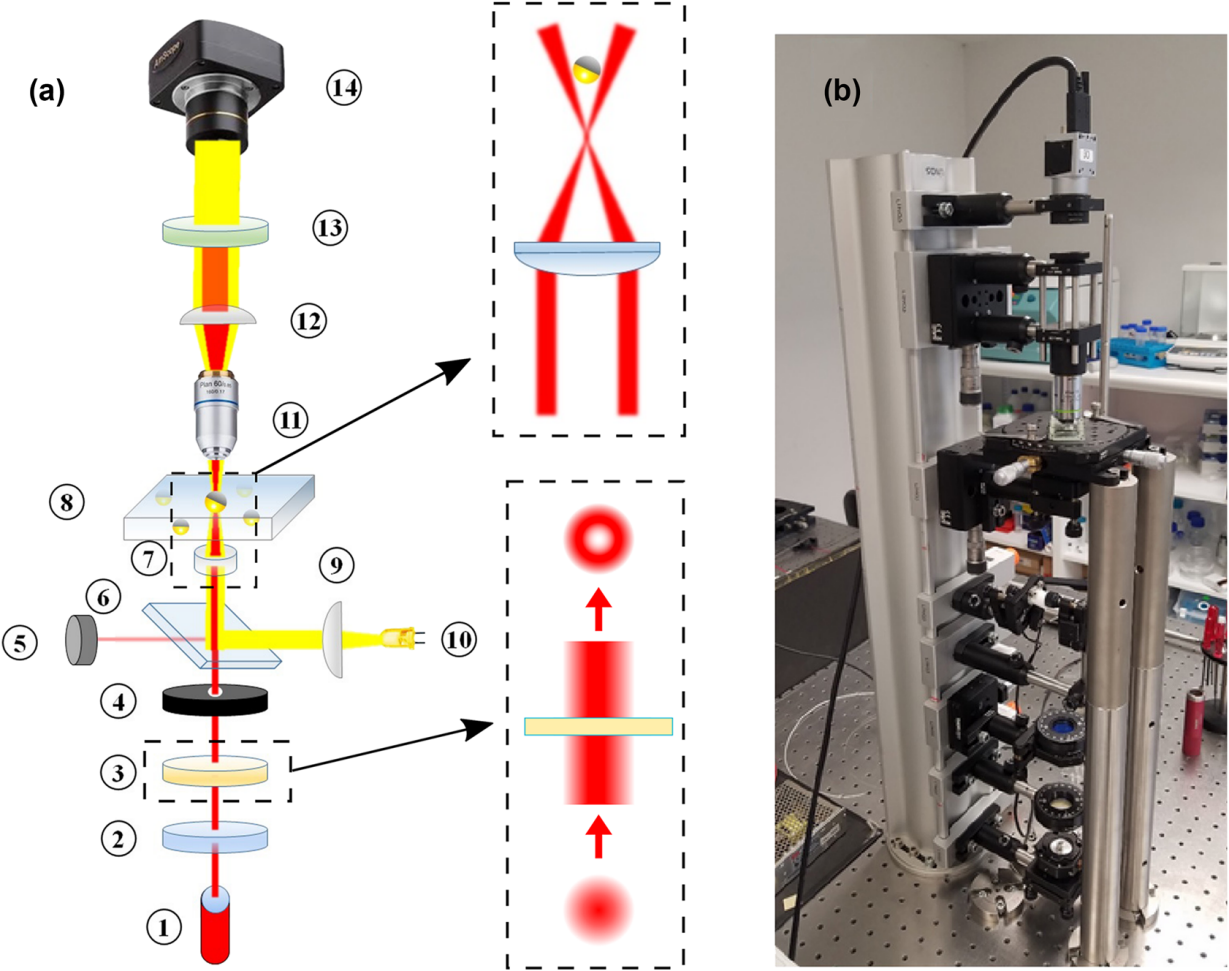
Experimental setup for the levitation of Janus particles: (a) Schematic representation of the optical setup. The top inset shows a sketch of 6 μm silica Janus particles with a 2 nm Ti and 50 nm Au cap levitated in a doughnut beam trap. For simplicity and clarity the doughnut beam is represented by it vertical central section. The bottom inset illustrate the generation of a CVB from a Gaussian beam using a the vortex phase-retarder. (b) Image of the assembled working setup.

We developed a setup based on a customized optical microscope to levitate and study Janus particles within focused cylindrical beams. The setup consists of two main parts. The imaging part, which includes the aforementioned optical microscope positioned above the sample plane, handles visualization and data processing. The levitation part, positioned below the sample plane, is responsible for converting an initial Gaussian beam into a cylindrical vector beam and then tightly focusing it onto the sample, as illustrated in Figure 3.

In the levitation part a laser source (1) (Thorlabs BL976-SAG300 – 976 nm) generates an upward-pointing Gaussian beam with a wavelength of *λ* = 976 nm. This beam passes through a linear polariser (2) (Thorlabs LPNIRE100-B) and then through a vortex phase-retarder (3) (Thorlabs WPV10L-980), which converts it into a beam with a doughnut-shaped intensity profile, see bottom inset of Figure 3(a). This beam has minimal intensity at its centre, and its polarisation can be adjusted from radial to azimuthal by rotating the optical axis of the vortex phase-retarder. To minimize reflections from optical elements and improve beam quality, a diaphragm (4) with a 1.5 mm aperture is placed just after the phase retarder (see Figure 3). A long-pass dichroic mirror (6) with a cut-on wavelength *λ* = 805 nm (Thorlabs DMLP805) is used to direct a 10 % of beam intensity onto a power meter (5) (Thorlabs S120C) to monitor power stability. Finally, an aspheric lens (7) with a focal length of *f* = 3.1 mm NA = 0.7 (Thorlabs C330TMD-B) focuses the beam onto the sample. The beam diameter is thereby reduced from 2 mm to approximately 3 μm, comparable to the size of the Janus microparticles. The aspheric lens is mounted on a vertical translation stage to control the focal spot’s vertical position and, consequently, the height of the levitated particle within the sample (8). The sample is held on a 2D precision translation stage (Thorlabs NFL5D/M), allowing precise positioning in the horizontal plane. This setup enables control over the levitation of Janus particles, which are dispersed in an aqueous solution. The imaging part includes an illumination source (10), an objective (11), a collimation lens (9) and digital camera (14). We use collimated white light LED (10) which is combined with the laser beam path using a long-pass dichroic mirror (6) to illuminate the region of interest. A 20X plan achromat objective (11) (Olympus RMS20X) projects the investigated area onto the digital camera for further data processing. The diverging beam after the objective is collimated using a plano-convex lens (12) with a focal length of *f* = 50 mm (Thorlabs LB1471-B). Finally, a pair of short-pass dichroic filters (13) (Thorlabs FGB37M and FGS900M) with bandpass ranges of 335–610 nm and 315–710 nm, respectively, is placed immediately before the digital camera (14) to block NIR radiation from reaching the camera.

To achieve effective and controlled levitation of a highly reflective Janus particle, it is necessary to balance all the forces described in Section 2. The Janus particles used during our investigation have a 50 nm and 140 nm thickness of the golden layer providing a high reflectivity *R* > 98 % of the Janus particles metallic cap.

To investigate the dependence of trap stiffness, which indicates how strongly a particle is trapped, on laser power, metallic cap thickness, and polarisation state (radial or azimuthal), we recorded the dynamics of trapped particles via digital video microscopy. Each experiment consisted of a 30,000-frame video recorded at 100 frames per second. The acquired videos were analysed with a MATLAB script based on digital video microscopy techniques [52] to extract particle trajectories and calculate the trap stiffness by the standard deviation calibration method [29], [31].

For statistical reliability, each experiment is repeated at least three times using different particles of the same type each time and the result shown here are the average of at least three measurements. Experiments are conducted within a laser power range of 0.2–80 mW. The experimentally determined threshold power for levitating the lightest Janus particle, a PS Janus particle by CVB with radial polarisation, is *P* = 0.5 mW. At powers exceeding 80 mW, the heaviest particles investigated are pushed to the upper surface of the sample.

## 4 Results

A laser beam with a wavelength of *λ* = 976 nm and with a doughnut intensity profile (with a central intensity minimum) is focused onto the sample from below, through the bottom substrate. At a certain threshold laser power, the radiation pressure becomes strong enough to lift the Janus particles in the *z* direction along the propagating beam and above the focal spot, to an equilibrium height, where the upward optical pressure is balanced by the gravitational force. Here, 2D lateral confinement is achieved using the surrounding ’bright’ walls of the doughnut beam, repelling a reflective particle towards the ’dark’ centre of the beam, as illustrated in the top inset of Figure 3(a).

**Figure 4:**
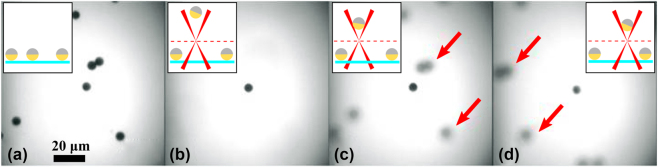
Levitation of Janus particles using a cylindrical vector beam: (a) Janus particles are initially resting on the bottom surface of the microscope slide. (b) A single Janus particle is lifted by radiation pressure and trapped within the dark centre of the doughnut beam. (c–d) The trapped particle is moved laterally across the sample plane to demonstrate effective levitation and manipulation. The red arrows are used as references, indicating the same particles in (c) and (d), emphasizing that the trapped particle moved to the right relative to them when the translational stage was moved to the left. The relative positions of the Janus particles on the bottom remain fixed, while their positions shift with respect to the trapped Janus particle. The insets show a lateral sketch of the entire process. The cyan line represents the bottom glass of the sample cell, while the dashed red line represents the focal plane.

Experimental evidence of levitation of a Janus sphere is shown in Figure 4(a)–(d). Here a 6 μm silica Janus particle with a 2 nm Ti and 50 nm Au cap located at the bottom of the sample glass (see Figure 4(a)) is levitated in an aqueous sample, reaching a height of 60 μm above the bottom substrate at a laser power of *P* = 5 mW, as showed in Figure 4(b). The particle was subsequently lowered to approximately 10 μm above the bottom substrate (see Figure 4(c)), making the shadows of nearby particles on the bottom visible for reference, and is then moved along the sample plane as shown in Figure 4(d). All particle manipulations are conducted using precise manual *x* − *y* − *z* translation stages. We will present results for four different types of Janus particles: Silica-50 nm gold cap, PS-50 nm gold cap, Silica-140 nm gold cap and PS-140 nm gold cap. Each type of particle will be analysed in two configurations: CVB azimuthal and CVB radial. For both the 50 nm and 140 nm gold caps, the gold layer is practically fully reflective. The primary difference between these cases lies in the increased weight of the thicker gold cap. The increased weight (due to the additional gold) brings the particle relatively closer to the focus, leading to higher optical intensity at the particle’s location. Consequently, particles with thicker gold caps experience greater absorption of optical power.

### 4.1 Effects of azimuthally polarised laser light on Janus particles

**Figure 5:**
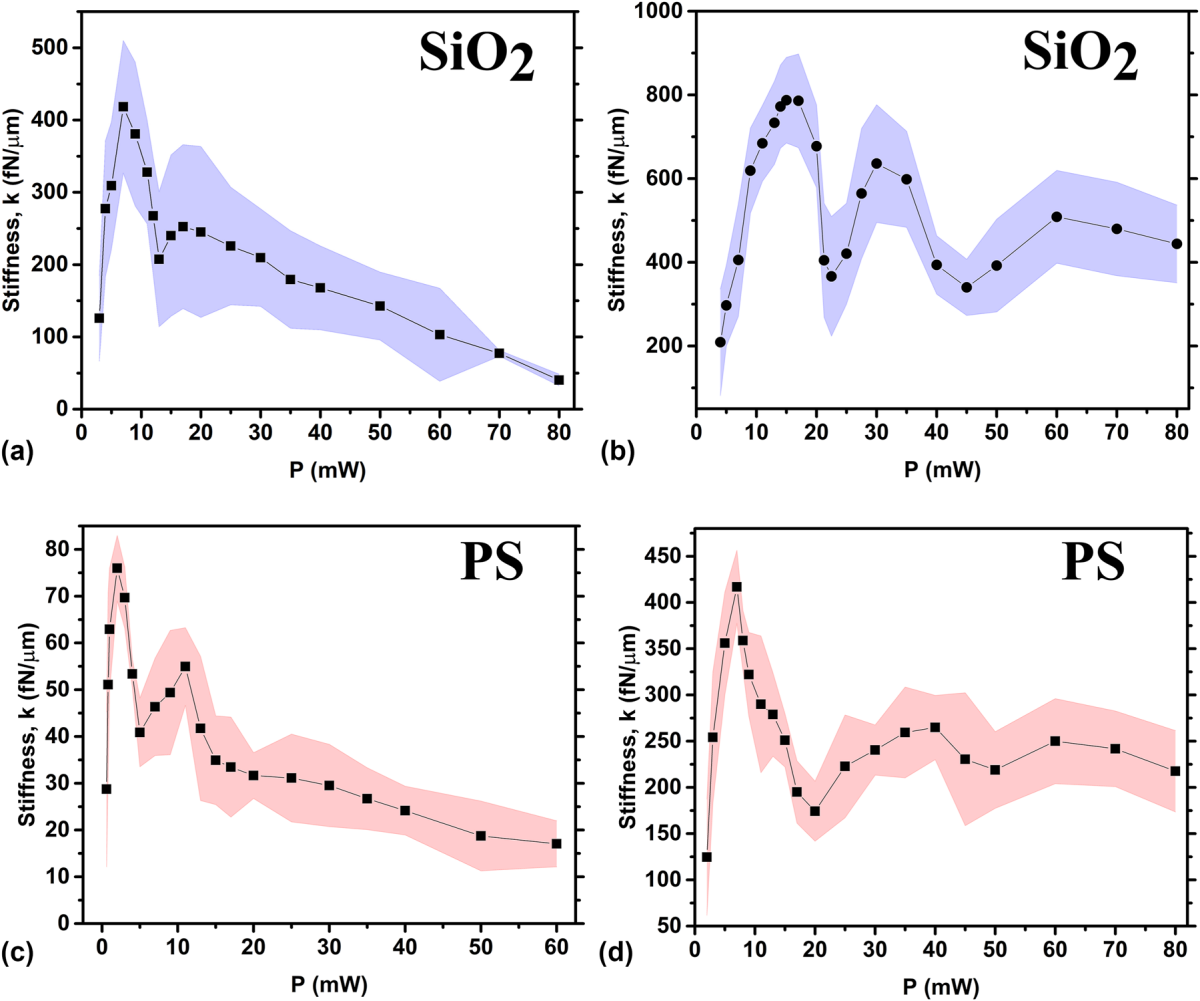
Trap stiffness as a function of applied laser power under azimuthally polarised beam placed within a glass slide with an internal spacing of 130 μm. (a) Trap stiffness of SiO_2_ Janus particles with 6 μm in size and coated with 2 nm Ti and 50 nm Au, and (b) 2 nm Ti and 140 nm Au. (c) Trap stiffness of PS Janus particles with 6 μm in size and coated with 2 nm Ti and 50 nm Au, and (d) 2 nm Ti and 140 nm Au cap. Black marks represent the stiffness values while the dashed areas represent the statistical error.

In contrast to the stiffness dependence observed for dielectric microparticles trapped by classical optical tweezers, where a linear relationship occurs, we find a complex behaviour in the trend of average stiffness $k=\frac{k_{x}+k_{y}}{2}$ versus applied laser power for 6 μm SiO_2_ Janus particles with a 2 nm Ti and 50 nm Au cap in a glass sample cell with an internal thickness of 130 μm under cylindrical vector beam trap, as shown in Figure 5(a).

It is noticeable that the threshold laser power required to levitate a SiO_2_ Janus particles under azimuthally polarised cylindrical vectors beam is *P*_min_ = 3 mW. As the laser power increases, the trap stiffness rapidly rises to a peak value, followed by a gradual decrease. However, a significant drop in stiffness is observed in the power range of 11–15 mW.

Comparing these results with equally-sized PS Janus spheres, levitated always by azimuthally polarised cylindrical vectors beam, we observe that the threshold power required for its levitation decreased to *P*_min_ = 0.6 mW, as shown in Figure 5(c). This is expected, given the more than twofold lower density of PS compared to SiO_2_ (*ρ*(PS) = 1.05 g/cm^3^, *ρ*(SiO_2_) = 2.65 g/cm^3^, according to the producer microParticles GmbH), thus reduces the radiation pressure required to lift particles with lower gravitational weight. From Figure 5(c), it is evident that the overall stiffness trend resembles that observed for SiO_2_ Janus particles, with a rapid increase to a peak value followed by a gradual decrease. In this case, a significant drop in stiffness is also observed; however, it shifted to a lower laser power range of 4–9 mW. It is noteworthy that the trap stiffness values for PS Janus particles are significantly smaller than those for SiO_2_. For instance, the peak stiffness for SiO_2_ Janus particles reached *k*_max_(SiO_2_) = 418 pN/μm, whereas for PS Janus spheres it only reach *k*_max_(PS) = 75 pN/μm. At the laser power *P* > 60 mW the gravitational weight of PS Janus particles is insufficient to counterbalance the radiation pressure, resulting in continuous upward particle motion until reaching the top boundary.

A possible intuitive explanation of this behaviour is that starting from a threshold laser power, when radiation pressure becomes sufficient to lift the Janus microparticles above the substrate, we typically observe a minimum in stiffness. At these low power levels, the intensity of the light cone walls surrounding the particle is not yet strong enough to forcefully repel the particle toward the “dark” centre. As a result, particles remain close to the focal spot, where the “dark” region is narrow. Under these conditions, thermal fluctuations allow Janus particles to penetrate relatively deeply into the bright region, resulting in weakly localized (broad) trajectories and thus low stiffness values. As laser power increases slightly, the particle’s height above the focal spot remains nearly unchanged, so the “dark” region continues to be narrow. However, the boundary intensity becomes more pronounced, leading to stronger repulsion of Janus particles toward the “dark” centre. Under these conditions, thermal fluctuations of the Janus spheres are more constrained, causing a rapid increase in trap stiffness. Beyond a certain optimal laser power (corresponding to the peak stiffness value shown in Figure 5(a) and (b)), further increases in power raise radiation pressure, causing the particle’s levitation height above the focal spot to increase. The particle quickly reaches equilibrium, and as the laser power increases, the “dark” region changes slightly. As the particle’s altitude increases, the beam broadens due to defocusing. This broadening of the optical potential wall allows particles to penetrate deeper, leading to a decrease in stiffness. Additionally, changes in particle weight influence levitation height above the focus. Lighter particles, such as PS, levitate higher above the focal spot than their SiO_2_ counterparts; thus, at the same beam power, trap stiffness is greater for SiO_2_ Janus spheres.

By increasing the thickness of the gold cap on 6 μm SiO_2_ Janus particles from 50 nm to 140 nm, while maintaining the Ti cap thickness at 2 nm, the Janus particles become heavier without significantly altering their reflectivity, which remains between 98 % and 99 %. This is because the penetration depth of light in the 50 nm Au cap is only 12 nm, which is much smaller than the cap thickness. For the 140 nm Au cap, the penetration depth remains the same at 12 nm, see Supplementary Information for more details.

Janus particles with a thick gold layer are particularly interesting for a detailed study, as they tend to be localized closer to the beam’s focal plane. Therefore, it is expected that these particles experience a significantly greater heating. Taking into account the results described above, one can expect a notable enhancement in trap stiffness due to the increased weight of the particles. As shown in Figure 5, peak trap stiffness values indeed increases for both SiO_2_ and PS Janus particles reaching approximately $k_{SiO_{2}}\approx$ 800 fN/μm and *k*_PS_ ≈ 425 fN/μm, respectively, see Figure 5(b) and (d). These stiffness values are reached at *P* = 15 mW for SiO_2_ and *P* = 7 mW for PS Janus particles. Interestingly, we observe the appearance of a second drop in the stiffness-versus-power trends, with minima at *P* = 45 mW and *P* = 50 mW for SiO_2_ and PS Janus particles, respectively. Furthermore, for the same power, particles levitate at different heights relative to the focus of the beam. This is confirmed by the inspection of Figure 5(a) and (d). The effective weight of SiO_2_ with a 50 nm Au cap is 1.38 pN, while that of PS with a 140 nm Au cap is 1.29 pN. At low power, the stiffness values are similar. However, the stiffness of PS Janus particles with a 140 nm Au cap is slightly lower than that of SiO_2_ particles with a 50 nm Au cap. This is because the levitation equilibrium distance of the PS particle is slightly higher. This observation holds only at low power and specifically for the azimuthal case. This is because the “centre” of the beam above the focus is “dark,” resulting in minimal absorption. Therefore, at low beam power, this explanation suffices to account for the observed similarity. So far, the non-monotonic behaviour of the stiffness after the first peak, as shown in Figure 5, is not entirely clear. It may depend on the presence of hydrodynamic or convective fluxes occurring in the chamber due to the beam, or it could be attributed to thermophoretic forces, given that the particle absorbs 2 % of the impinging optical power.

### 4.2 Effects of radial polarised laser light on Janus particles

**Figure 6:**
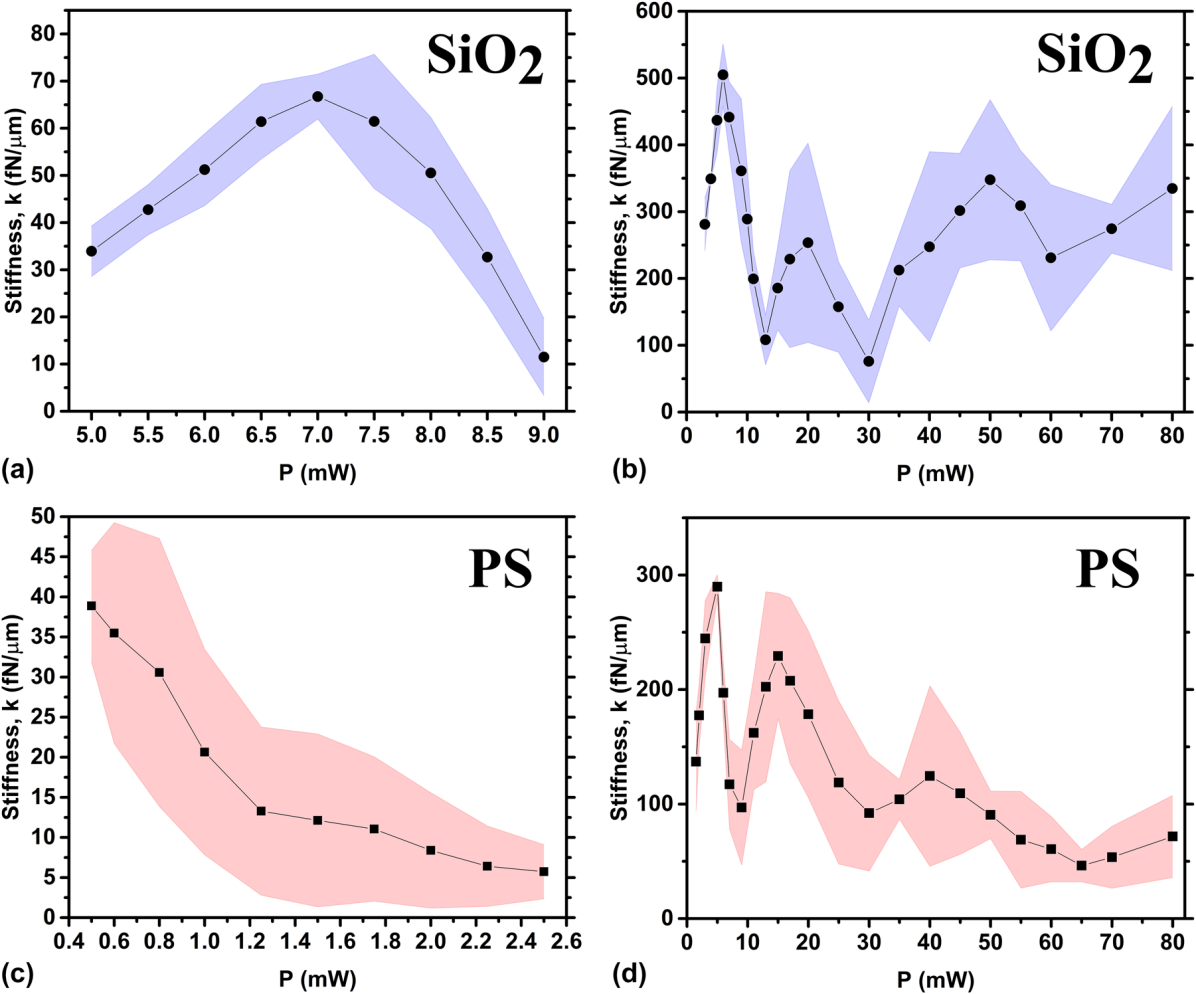
Average stiffness *k* for different values of the levitation power *P* for 6 μm Janus particles with a 2 nm Ti cap made of: (a) SiO_2_ with 50 nm Au cap; (b) SiO_2_ with 140 nm Au cap; (c) PS with 50 nm Au cap; (d) PS with 140 nm Au cap under radially polarised beam.

The optical properties of radial polarised cylindrical vector beams differ considerably from those of azimuthally polarised beams, indeed, according to Maxwell’s equations, azimuthally polarised cylindrical vector beams have only a transverse field component [53]. In particular, above the focal spot, this results in a pronounced contrast between the doughnut-shaped beam walls and the “dark” central hole. However, switching to a radially polarised beam state redistributes the optical field, introducing an additional longitudinal component. This longitudinal field reduces the contrast between the beam’s “dark” centre and its “bright” walls, destabilizing the trapped Janus particles. Indeed, for both SiO_2_ and PS Janus particles with a 2 nm Ti and 50 nm Au cap the average stiffness *k* shows a significant reduction and a narrowing of the working laser power range, as shown in Figure 6(a) and (c) compared to the previous case with azimuthal polarisation. In particular, we find that for SiO_2_ Janus particles, levitated by a radial polarised cylindrical vector beam, the peak stiffness value *k* ≈ 65 fN/μm is observed at *P* = 7 mW and the total working range shrinks to 5 mW ≤ P≤ 9 mW, see Figure 6(a). Similarly, we can trap PS Janus particles in a smaller applied laser power range 0.5 mW ≤ P≤ 2.5 mW with a peak stiffness value *k* ≈ 40 fN/μm measured at *P* = 0.5 mW, see Figure 6(b). Naturally, in such a narrow range of levitation powers we do not observed any rapid drop of the stiffness trend. Moreover, in this case the stiffness trend behaviour inherent to the radially polarised beams is not evident. In order to stabilize Janus particles within a trap and extend a working laser power range, we increase the thickness of the deposited Au cap layer to 140 nm. As we expect it allow us to trap both SiO_2_ and PS Janus particles in a wide range of powers up to *P* = 80 mW, like it is shown in Figure 6(b) and (d). Unexpectedly we find very complex stiffness trends behaviour with a peak stiffness values $k_{SiO_{2}}\approx$ 500 fN/μm at *P* = 7 mW and *k*_PS_ ≈ 300 fN/μm at *P* = 5 mW for SiO_2_ and PS Janus spheres respectively. The obtained maximal stiffness values are notably smaller comparing to data measured in azimuthally polarised beams, proving the assumption that greater contrast between “dark” hole and “bright” walls results in more effective levitation of highly-reflective Janus microparticles. Besides, under such conditions we observe the appearance of three notable drops in the stiffness trends (see Figure 6(b) and (d)). We suppose that within radially polarised beams Janus particles might absorb additional radiation energy from longitudinal component of the optical field, resulting in increasing of thermal fluctuations.

## 5 Conclusions

In this work we investigate the behaviour of Janus particles under focused cylindrical vector beams, demonstrating a controlled levitation mechanism harnessing the distinct optical and thermodynamic properties of Janus particles. By systematically varying parameters such as laser power, metallic cap thickness, and polarisation state, we observe different trend of the levitation stiffness *k*. In particular, gravity, radiation pressure, beam diameter at the particle plane, and hydrodynamic fluxes are the primary forces influencing the behaviour of trap stiffness. Janus particles manipulation is crucial for fields such as nanotechnology, biophysics, and environmental science, where Janus particles can function as micro-tools, targeted drug delivery vehicles, or microscale sensors [54], [55]. The ability to control Janus particles with precision paves the way for applications in micro-robotics, where Janus particles could serve as self-propelling micromachines, and in diagnostic technologies, where their selective responsiveness to environmental stimuli could enable localized sensing or targeted therapeutic delivery [55], [56], [57].

## Supplementary Material

Supplementary Material Details
