# Supplementary material for: Optical levitation of Janus particles within focused cylindrical vector beams
Source: Nanophotonics. 2025 Apr 28;14(23):4113–24. doi: 10.1515/nanoph-2024-0774 (PMC12617708; doi:10.1515/nanoph-2024-0774)
Supplement: Supplementary file 1 — Supplementary Material Details [file j_nanoph-2024-0774_suppl_001.pdf]

Alessandro Magazzù*, Iryna Kasianiuk, Denis Kasyanyuk, Agnese Callegari, Giovanni Volpe, Onofrio M. Maragò, and Luca Biancofiore

Supplementary Information

Additional information

We list below the needed additional information to (i) justify our assumptions and (ii) help with the interpretation of the experimental data.

Optical properties of the gold cap

Here we list the properties of a gold film of different thickness for a reference optical wavelength in vacuum of $\lambda_0 = 976\,\text{nm}$. We list in Table 1 the reference values of the complex refractive index $\tilde{n} = n - ik$, the absorption coefficient $\alpha = 4\pi k/\lambda_0$, the penetration depth $\delta_\text{p} = 1/\alpha$, and of the non-polarized reflectance R obtained from different references in the literature. Inspecting it is evident that, for the cap thickness that are used in our experiments, the precise reference used for determining the optical properties does not matter much (i.e., the values are practically equivalent). In fact, the reflectance is in all cases $R \approx 98 - 99\%$. Therefore, when Janus particles with cap of different thickness are subject to the same optical field, we can assume that they feel the same optical force (same scattering force, same gradient force), same amount of absorbed optical power P_abs.

***Corresponding author: Alessandro Magazzù,** CNR-IPCF, Istituto per i Processi Chimico-Fisici, I-98158, Messina, Italy, alessandro.magazzu@cnr.it;
Iryna Kasianiuk, Department of Mechanical Engineering, Bilkent University, TR-06800, Ankara, Turkey; UNAM - National Nanotechnology Research Center and Institute of Materials Science & Nanotechnology, Bilkent University, 06800 Ankara, Turkey
Denis Kasyanyuk, Department of Mechanical Engineering, Bilkent University, TR-06800, Ankara, Turkey; UNAM - National Nanotechnology Research Center and Institute of Materials Science & Nanotechnology, Bilkent University, 06800 Ankara, Turkey
Agnese Callegari, Department of Physics, University of Gothenburg, SE-41296, Gothenburg, Sweden;
Giovanni Volpe, Department of Physics, University of Gothenburg, SE-41296, Gothenburg, Sweden;
Onofrio M. Maragò, CNR-IPCF, Istituto per i Processi Chimico-Fisici, I-98158, Messina, Italy;
Luca Biancofiore, Department of Mechanical Engineering, Bilkent University, TR-06800, Ankara, Turkey; UNAM - National Nanotechnology Research Center and Institute of Materials Science & Nanotechnology, Bilkent University, 06800 Ankara, Turkey; University of L'Aquila, L'Aquila, Italy;

Mass of the Janus particles

In the experiments, we use four different kinds of Janus particles:

- SiO_2(6 µm)@Au(50 nm),
- PS(6 µm)@Au(50 nm),
- SiO_2(6 µm)@Au(140 nm),
- PS(6 µm)@Au(140 nm)

where with the notation A(d)@B(h) we mean a Janus particle composed by a bare particle of material A with size d with a coating layer of material B with thickness h.

Such particles differ in their mass, gravitational weight, and buoyancy only, as the optical properties of their coating layer does not depend on its thickness in this specific case (see Table 2 of the optical properties of the coating layer).

Summarizing:

Particle	SiO_2@Au	PS@Au	SiO_2@Au	PS@Au
Layer thickness h	50 nm	50 nm	140 nm	140 nm
Effective Weight	1.38 pN	0.40 pN	2.18 pN	1.29 pN

The reference values above have been calculated using $\rho_{\text{SiO}_2} = 1.85 \times 10^3\,\text{kg/m}^3$, $\rho_\text{PS} = 1.05 \times 10^3\,\text{kg/m}^3$, $\rho_\text{Au} = 17 \times 10^3\,\text{kg/m}^3$.

Power absorption and temperature calculation of the Janus particles

The zero-order vortex retarder used in our experiments generates radial or azimuthal polarised beams, depending on the rotation of the optical axis. Since in our experiment, a 6 μm SiO_2 with a 50 nm thick gold cap is levitated approximately 50 μm above the focal spot, we can consider both beams with a doughnut intensity profile and an average power of $P = 50$ mW. Thus, in order to calculate the intensity profile impinging on the JP particle and the power absorption, we can use a paraxial approximation. The equivalent beam waist ω_0^CVB at the focal plane for a doughnut radial/azimuthal beam can be expressed as:

Tab. 1: Reference values of the complex refractive index $\tilde{n} = n - ik$, the absorption coefficient α, the penetration depth $\delta_{\rm p}$, and of the non-polarized reflectance R obtained from different references in the literature.

Reference used	[1]	[1]	[2]	[3]
Thickness h	53 nm	117 nm	140 nm	140 nm
n	0.20745	0.16623	0.1506	0.17111
k	6.6332	6.5248	6.4258	6.4613
Absorption coefficient α	8.54×10^5 cm^{-1}	8.4×10^5 cm^{-1}	8.27×10^5 cm^{-1}	8.32×10^5 cm^{-1}
Penetration depth $\delta_{\rm p}$	11.7 nm	11.9 nm	12.1 nm	12.0 nm
Reflectance (non-polarized) R	0.98	0.98	0.99	0.98

Tab. 2: Calculation of gravitational weight and buoyancy for different Janus particles.

Particle	SiO_2@Au	PS@Au	SiO_2@Au	PS@Au
Layer thickness h	50 nm	50 nm	140 nm	140 nm
Bare particle mass $m_{\rm bare}$	209.23×10^{-15} kg	118.76×10^{-15} kg	209.23×10^{-15} kg	118.76×10^{-15} kg
Cap mass $m_{\rm cap}$	48.07×10^{-15} kg	48.07×10^{-15} kg	134.59×10^{-15} kg	134.59×10^{-15} kg
Total mass $m_{\rm JP}$	257×10^{-15} kg	157×10^{-15} kg	344×10^{-15} kg	253×10^{-15} kg
Weight $w_{\rm JP}$	2.52 pN	1.54 pN	3.37 pN	2.48 pN
Total volume $V_{\rm JP}$	116 μm^3	116 μm^3	121 μm^3	121 μm^3
Buoyancy $F_{\rm buoy}$	1.14 pN	1.14 pN	1.19 pN	1.19 pN
Effective Weight	1.38 pN	0.40 pN	2.18 pN	1.29 pN

$$\omega_0^{\rm CVB} = \sqrt{2}\omega_0 \quad (1)$$

where ω_0 is the beam waist of a fundamental Gaussian beam [4], which can be calculated as [5]:

$$\omega_0 \cong \frac{\lambda}{\pi \rm NA} = \frac{0.976}{\pi\ 0.7} = 0.444\ \mu\rm m \quad (2)$$

So that $\omega_0^{\rm CVB} = 0.628\ \mu$m. On the other hand, the Rayleigh length for a doughnut beam is:

$$z_R \cong \frac{\pi {\omega_0^{\rm CVB}}^2}{\lambda} = \frac{\pi(0.628)^2}{0.976} = 1.27\ \mu\rm m \quad (3)$$

Thus, the beam waist of a doughnut at a given distance z from the focal plane in paraxial approximation is:

$$\omega(z) = \omega_0^{\rm CVB}\sqrt{1+\left(\frac{z}{z_R}\right)^2} \quad (4)$$

For $z = 50\ \mu$m we obtain $\omega = 24.754\ \mu$m. According to reference [6] the expression of the annular amplitude function can be written as:

$$A(r) \cong \sqrt{\frac{2}{\pi}}\frac{\sqrt{2}r}{\omega^2}\exp\left(-\frac{\rm r^2}{\omega^2}\right) \quad (5)$$

where r is the radial polar coordinate. Considering the intensity as the squared modulus of the amplitude function $I = |A(r)|^2$ we obtain:

$$I(r) = \frac{4r^2}{\pi\omega^4}\exp\left(-\frac{2\rm r^2}{\omega^2}\right) \quad (6)$$

Using a Phyton script, we compute the intensity profile of the doughnut beam in polar coordinates (r, ϑ) and normalize its double integral to $P = 50$ mW:

$$P = \int_0^{2\pi}\int_0^{r} I(r,\vartheta) r\ dr\ d\vartheta = 50\ \rm mW \quad (7)$$

Where ϑ is the angular coordinate. Using the same script, we also calculate the double integral over a circular region corresponding to the area of the JP, as shown in Figure 1, where the integration area is represented by the black circle. As an example, we assume that the JP il located between the regions of highest and lowest intensity, randomly fluctuating within the annular intensity profile. In this position the integrated power impinging normally on the JP is $P_{\rm imp} \cong 0.32$ mW. This is an approximation, as it

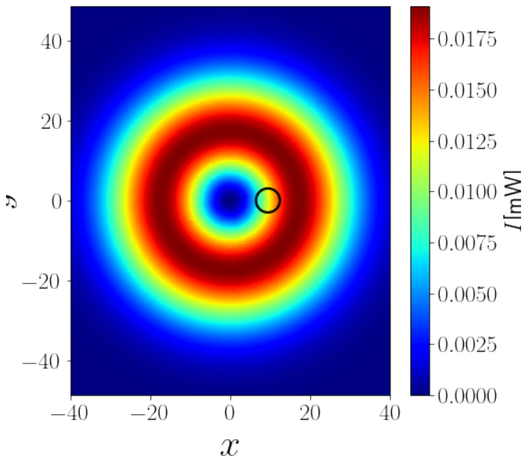

Fig. 1: Intensity profile $I(r, \vartheta)$ in cartesian coordinates of a doughnut beam, where the black circle represents the JP area, over which the double integral is performed.

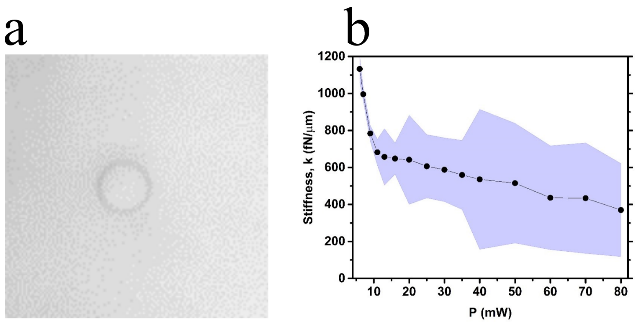

Fig. 2: a) Uncoated dielectric 6 μm SiO_2 microsphere suspended in water and levitated by a CVB laser beam with azimuthal polarisation. b) Average stiffness k for different values of the levitation power P for a 6 μm plain SiO_2 microsphere.

assume that the intensity profile does not depends on the incident angle θ with the JP gold shell. Conversely, considering $I(\theta)$, the power impinging on the JP can be approximated as:

$$P \cong \int_{ill.surf.} I(\theta)\ \cos(\theta)\ [R(h,\theta)\ \cos(\theta) + A(h,\theta)\ ds] \tag{8}$$

where ds is he differential surface element, the integral is performed on the illuminated surface and $\cos(\theta)$ accounts for the projection of the local surface normal onto the beam axis, $A(h, \theta)$ is the absorbance function of the gold, h is the thickness of the gold layer [1]. Sticking with our approximation (normal incident) we set $\theta = 90°$ and $h = 50$nm according to one of the two different type of JP used during our experiments, then $A(50, 90°) = 0.02$ and the power absorbed by the JP can be approximated as:

$$P_{\rm abs} \cong P_{\rm imp} \cdot A(h,\theta) = 0.32\ \mu{\rm W} \cdot 0.02 = 6.4\ \mu{\rm W} \tag{9}$$

Then, the increase of temperature ΔT at the particle cap, according to Eq. 6 of the manuscript is:

$$\Delta T \cong \frac{P_{\rm abs}}{(\pi + 2) k_m d} = 0.35\ {\rm K} \tag{10}$$

Radiation pressure

The scattering forces due to radiation pressure depend primarily on the thickness of the gold cap and in the case of no normal incidence can be expressed as:

$$F_{\rm rp}(h,\theta) \cong \frac{1}{c} \int_{ill.surf.} I(\theta)\ \cos(\theta)\ [R(h,\theta)\ \cos(\theta) + A(h,\theta)\ ds] \tag{11}$$

Control experiment

We performed control experiments on 6 μm plain SiO_2 particles under azimuthal polarisation. In particular, we observed that dielectric particles at small applied laser powers are levitated in the center of the beam. The stiffness decreases monotonically as the power increases as shown in Figure 2. This is because, on one side the low numerical aperture is not enough to grant trapping in the axial direction, so we still have levitation rather than trapping. On the other side, a higher power yields a higher radiation pressure, a larger levitation effect, which in turn brings the JP in a region with a broader doughnut beam profile, thus reducing the optical gradient force. The monotonically behaviour of the trap stiffness for plain SiO_2 particles contrast with the intriguing non monotonic stiffness behaviour observed for the JPs and highlights that heating due the gold cap plays a dominant role in the optical levitation of JP.

References

[1] D. I. Yakubovsky, A. V. Arsenin, Y. V. Stebunov, D. Yu. Fedyanin, and V. S. Volkov, "Optical constants and structural properties of thin gold films," *Opt. Express*, vol. 25, no. 21, pp. 25574–25587, 2017.

[2] R. L. Olmon, B. Slovick, T. W. Johnson, D. Shelton, S.-H. Oh, G. D. Boreman, and M. B. Raschke, "Optical dielectric function of gold," *Phys. Rev. B*, vol. 86, p. 235147, 2012.

[3] M. Magnozzi, M. Ferrera, L. Mattera, M. Canepa, and F. Bisio, "Plasmonics of Au nanoparticles in a hot thermodynamic bath," *Nanoscale*, vol. 11, pp. 1140–1146, 2019.
[4] T. Liu, et al., "Research on the intensity profiles of high-order Laguerre-Gaussian mode laser beams," Infrared Phys. Technol., vol. 141, p. 105459, 2024.
[5] P. H. Jones, O. M. Maragò, and G. Volpe, "Optical tweezers: Principles and applications," Cambridge: Cambridge University Press, 2015.
[6] R. Li, et al., "Generating large topological charge Laguerre–Gaussian beam based on 4K phase-only spatial light modulator," Chin. Opt. Lett., vol. 20, no. 12, p. 120501, 2022.
